# mRNA-Based Vaccine Designing against Epstein-Barr Virus to Induce an Immune Response Using Immunoinformatic and Molecular Modelling Approaches

**DOI:** 10.3390/ijerph192013054

**Published:** 2022-10-11

**Authors:** Hassan N. Althurwi, Khalid M. Alharthy, Faisal F. Albaqami, Ali Altharawi, Muhammad Rizwan Javed, Ziyad Tariq Muhseen, Muhammad Tahir ul Qamar

**Affiliations:** 1 Department of Pharmacology and Toxicology, College of Pharmacy, Prince Sattam Bin Abdulaziz University, Al-Kharj 11942, Saudi Arabia; 2 Department of Pharmaceutical Chemistry, College of Pharmacy, Prince Sattam Bin Abdulaziz University, Al-Kharj 11942, Saudi Arabia; 3 Department of Bioinformatics and Biotechnology, Government College University Faisalabad (GCUF), Faisalabad 38000, Pakistan; 4 Department of Pharmacy, Al-Mustaqbal University College, Hillah 51001, Babylon, Iraq

**Keywords:** reverse vaccinology, Epstein-Barr Virus, multi-epitopes mRNA vaccine, molecular docking, immune simulations

## Abstract

Epstein-Barr Virus (EBV) is a human pathogen that has a morbidity rate of 90% in adults worldwide. Infectious mononucleosis is caused by EBV replication in B cells and epithelial cells of the host. EBV has also been related to autoimmune illnesses, including multiple sclerosis and cancers like nasopharyngeal carcinomas and Burkitt’s lymphoma. Currently, no effective medications or vaccinations are available to treat or prevent EBV infection. Thus, the current study focuses on a bioinformatics approach to design an mRNA-based multi-epitope (MEV) vaccine to prevent EBV infections. For this purpose, we selected six antigenic proteins from the EBV proteome based on their role in pathogenicity to predict, extract, and analyze T and B cell epitopes using immunoinformatics tools. The epitopes were directed through filtering parameters including allergenicity, toxicity, antigenicity, solubility, and immunogenicity assessment, and finally, the most potent epitopes able to induce T and B cell immune response were selected. In silico molecular docking of prioritized T cell peptides with respective Human Leukocytes Antigens molecules, were carried out to evaluate the individual peptide’s binding affinity. Six CTL, four HTL, and ten linear B cell epitopes fulfilled the set parameters and were selected for MEV-based mRNA vaccine. The prioritized epitopes were joined using suitable linkers to improve epitope presentation. The immune simulation results affirmed the designed vaccine’s capacity to elicit a proper immune response. The MEV-based mRNA vaccine constructed in this study offers a promising choice for a potent vaccine against EBV.

## 1. Introduction

Epstein-Barr Virus is a member of the herpes viruses γ family. It has a linear dsDNA genome (approximately 171 Kb) that codes for around 90 genes [1]. The EBV genes are categorized into immediate early genes, early genes, and late genes based on their expression. In the lytic life cycle of EBV, the immediate early genes that code for transcription factors are expressed very early, the early genes interfere with DNA synthesis and metabolism of the host, and the late genes code for structural and non-structural proteins [2]. EBV is further classified into two primary subgroups (type 1 and type 2); these two groups differ mainly in the EBNA-3 (nuclear antigen-3 gene). Both subgroups are reported worldwide; however, in most of the populations, type 1 is dominant [3]. EBV type 1 and 2 were initially distinguished on the basis of variations in the EBNA2. It has been reported that EBV-1 has a relatively longer EBNA-2 ORF (Open Reading Frame) than the EBV-2. In particular, when comparing EBV-1 and EBV-2 strains, EBNA-2 only has 70% gene homology, whereas it has 54% protein homology [4].

EBV is found in nearly 90% of the world’s adult population [5]. EBV causes an asymptomatic condition that frequently occurs during infancy in underdeveloped nations; however, in well-developed countries, the acute EBV infection is delayed for years until early adulthood with around 25% of infections developing into infectious mononucleosis (IM), which manifests as swollen lymph nodes, a high temperature, extreme fatigue, immunological dysfunction, and a sore throat [6]. EBV is the most common cause of IM and has also been linked to numerous cancers in humans such as stomach cancer, Hodgkin’s lymphoma, nasopharyngeal carcinoma, some entities of B cell and T cell lymphomas, and Burkitt’s lymphoma. Autoimmune illnesses including multiple sclerosis have also been linked to EBV [7,8,9]. The ability to promote cancer is facilitated by EBV’s strong growth transforming abilities. Virus particles are transferred from infected people’s saliva via close contact, entering the oral cavity, where it replicates via the lytic cycle in the oropharyngeal permissive cells. The viral particles are then shed into the pharynx in large volumes, followed by B cell infection, resulting in a long-term latent B cell infection [10,11].

The immune system can control the primary infection and then the reactivation phases of EBV, forcing it to remain latent in the memory of the B cells. There is currently no pharmacological cure for EBV, and no preventative or therapeutic vaccinations are available. Many efforts to develop a vaccine aimed at preventing EBV infection, have focused on the antigenic proteins, notably gp-350 because it is the primary target of neutralizing antibodies and is the amplest gp found on the viral particles [12,13]. Several vaccine trials were conducted between 2006 and 2015, including a candidate vaccine in a phase two trial based on the gp-350 protein that decreased the risk of IM, but not the viral infection [14,15]. This weakness can be attributed to differences in the characteristics of the vaccine such as effectiveness and the duration of protection offered against EBV infection. According to earlier research, most potential vaccines have a short lifetime, meaning that they might delay the infection, but may not be able to prevent long-term increases in IM infections [16]. Given the short period of protection provided by gp-350-based vaccines, a more efficient vaccine is needed that can offer long-term protection when used as a single or double dose [15]. Another reason that could explain the low efficiency of prospective EBV vaccines is the virus’s capability to circumvent immune responses. While most vaccines target gp-350, antigens that play critical roles in immune evasion have been discovered, such as BGLF5, BZLF1, BNLF2a, BILF1, EBNA1, and EBNA2 [17,18].

Conventional vaccines, such as inactivated and attenuated pathogens, provide long-lasting immune protection against the subject pathogen, but have limited large-scale and quick production protocols [19]. Because of their safety, relatively low production costs, rapid production capability, and improved efficacy, the rapidly emerging domain of mRNA therapies can be a powerful platform for addressing these issues. Furthermore, spontaneous degradation and a flexible half-life provide the significant safety advantage of the mRNA vaccine [20]. The mRNA comes with some inherent weaknesses, i.e., instability and inefficiency in delivery; however, recent advances in mRNA synthesis and modification through structural alterations have addressed these issues, offering promising results [21]. Bioinformatics-based vaccine development has offered an alternative approach with lower production costs and a largely reduced time required for developing the vaccines against cancer cells and infectious pathogens, in particular. Immunoinformatics approaches for vaccine development are being increasingly adopted after the recent COVID-19 pandemic; and many immunoinformatics studies have provided candidate vaccines to prevent a number of pathogens, including HIV-1, HSV-1 & 2, Ebola virus, norovirus, *Shigella* spp., and *Staphylococcus aureus* [22,23].

In our study, B and T cell epitopes that were predicted and filtered, were extracted from EBV proteins that were engaged in the self-assembly of the capsid, replication of DNA, and attachment of viral particles to the oral epithelial cells, were joined to construct a candidate mRNA vaccine against EBV. Furthermore, T cell epitopes were modeled for docking with respective HLA molecules to analyze their binding. HLA genes are highly polymorphic in humans, which presents a difficult challenge for designing a vaccine given that HLA molecules can bind a particular set of peptides [24,25]. The capacity to bind homologous peptides has been used to categorize most HLA-I alleles, with this categorization covering approximately 80 percent of the HLA-A and HLA-B alleles. Our findings from this study will be helpful for the development of an mRNA-based vaccine to provide immune protection against EBV.

## 2. Methodology

### 2.1. Collection of Proteins

**Figure 1:**
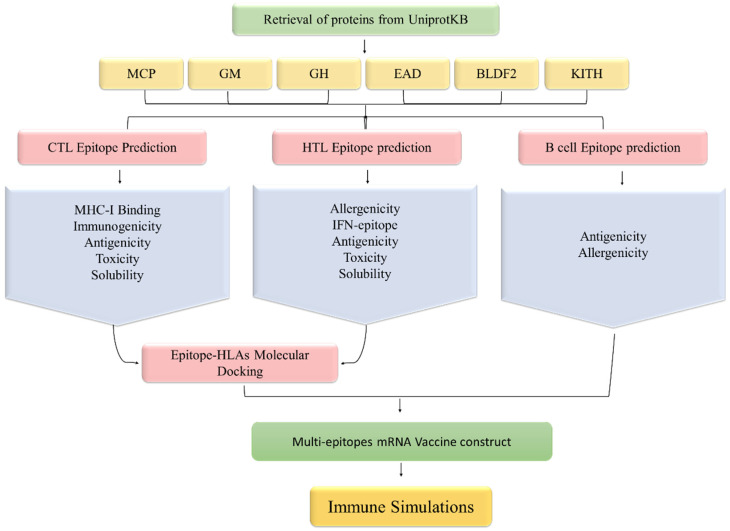
Methodological workflow of designing an mRNA vaccine against EBV in this study.

Six proteins (Major caps protein, Envelope glycoprotein M, Envelope glycoprotein H, DNA polymerase processivity factor BMRF1, Protein BDLF2, and Thymine kinase) were selected for this study based on their role in EBV infection and their attachment to host cells. The selected EBV proteins were downloaded from the Uniprot database [26]. The candidate protein’s antigenicity was calculated by the Vaxijen server [27]. The workflow of our study is provided in Figure 1.

### 2.2. MHC-I and MHC-II Binding Epitopes Prediction

The selected candidate proteins were used for epitopes prediction using online servers. NetCTL1.2 server was used at a 0.75 threshold to predict MHC I binding epitopes [28]. The server’s prediction is based on multiple calculations that include the binding of the epitope to MHC-I, proteasomal C-terminal cleavage, and transport efficacy of the Transporter Associated with Antigen Processing (TAP). The artificial neural network (ANN) calculates the binding of the peptide to the MHC-I receptor and also the Proteasomal C-terminus cleavage scores. The weight matrix is used to calculate TAP scores [28]. The NetCTL-1.2 technique has a sensitivity of over 0.80 among the 5% of top-scoring peptides in large standard calculations comprising 216 identified HIV epitopes that cover all twelve HLA supertypes.

The HTL epitopes prediction was made by the Immune Epitope Database (IEDB) [29], where a set of seven HLAs were taken as the reference set and all other parameters were taken as default in order to achieve maximum accuracy. HLAs are very polymorphic from person to person, and currently there is no broad screening system to ascertain if a link between the EBV’s incidence and the different HLA alleles exist, so it is more reasonable to set a reference set of HLAs that has the largest population coverage [30,31]. The server calculates IC50 values for predicted HTL epitopes, the IC50 value is inversely proportional to the epitope binding affinity to the MHC-II receptor. An IC50 value that is <50 nM is related to a higher binding affinity. An IC50 value that is <500 nM is related to an average binding affinity; whereas, an IC50 value that is <5000 nM is related to lower binding affinity. The server calculates a percentile rank that is inversely related to an epitope’s binding affinity to the MHC-II receptor. Multiple parameters were set to identify the epitopes fulfilling all the set criteria that would be further used for designing the mRNA vaccine [32].

### 2.3. Prioritization of T Cell Epitopes

Antigenicity. The Vaxijen server [27] was employed to predict the antigenic potential of the MHC I and MHC II epitopes that were predicted previously. The server calculates an antigenicity score based on the physiochemical properties of residues as a replacement for the alignment algorithm. The output results have a precision of 70–89% as reported by the authors of the Vaxijen server. Antigenic epitopes having a >0.4 score were selected for further analysis.

Immunogenicity. It is the potential of MHC I-binding epitopes to elicit a cell-mediated and humoral immune response. The MHC-I Immunogenicity calculator of the IEDB database [33] predicted the immunogenic property of the CTL epitopes. Immunogenic scores higher than zero were considered for more analysis.

Solubility. The antigenic and immunogenic epitopes that were shortlisted from previous steps were investigated for solubility by the CamSol Intrinsic server [34].

Toxicity. To make sure that we selected the non-toxic T cell epitopes only, the ToxinPred server was used to assess the toxicity of T cell epitopes. The server evaluates different physiochemical properties (Hydrophobicity, Amphipathicity, Hydropathicity, Hydrophilicity, pI, Net Hydrogen Charge, and Molecular weight) of an epitope to assess its toxicity [35]. The non-toxic and MHC I and MHC II epitopes fulfilling the aforementioned criteria were selected for further analysis.

MHC-I binding. MHC-I alleles for the prioritized CTL epitopes were predicted by the IEDB server (http://tools.iedb.org/mhci/) accessed on: 15 December 2021 [36].

Allergenicity. HTL epitopes that passed through the previous parameters were analyzed by the Algpred2.0 server for allergenicity [37]. Algpred2.0 has additional features to improve the working and accuracy of allergenicity prediction of the epitopes as compared to the previous version of the server developed in 2006. The allergenicity score was calculated with a sensitivity and specificity of 93% and 95%, respectively. The parameters were set at a cut-off score (0.3) to attain maximal accuracy. The HTL epitopes which were classified as non-allergenic passed to the next step of this prioritization protocol.

IFN gamma analysis. To predict the IFN-gamma-inducing ability of the prioritized HTL epitopes we used the IFN-epitope server developed by the Raghava group [38]. The IFN gamma uses SVM hybrid algorithms and motif techniques to calculate an SVM score for every epitope provided.

Population coverage. T cell epitopes filtered through the aforementioned steps were further analyzed for population coverage, globally. Using the default parameters at the IEDB population coverage module, the selected T cell epitopes were analyzed (both country-wise and regions-wise) to make sure that the selected epitopes used to construct the vaccine would cover most of the world’s population [39]. Regarding the distribution of their MHC-binding alleles, this tool is used to determine the average coverage of epitopes in different populations, and because EBV infections are reported worldwide a global analysis has been carried out.

### 2.4. Linear B Cell Epitope Prediction and Assessment

Linear B cell epitopes were predicted for the candidate proteins and subsequently evaluated for antigenicity and allergenicity. B cell epitopes bind to B cell receptors present on the surface of B cells. B cells are critical for antibody production by the immune system as the linear B cell epitopes induce the B cell response. BCPreds [40] predicts linear B cell epitopes for a given protein sequence based on the kernel method. The support vector machine (SVM) is a known element of kernel methods, and the SVM contains several algorithms for pattern examination. The performance of the aforementioned server (AUC = 0.75) is based on the SVM used in combination with residues of the pair antigenic property (AUC = 0.7). The AAP was utilized to predict linear B cell epitopes (LBL). The LBL epitopes were evaluated for allergenicity and antigenicity using the Algpred2.0 (cut-off = 0.3) and Vaxijen (cut-off = 0.4) servers, respectively. The prioritized LBL epitopes were joined together with the CTL and HTL epitopes.

### 2.5. Molecular Docking of T Cell Epitopes and HLAs

The selected T cell epitopes fulfilling the set parameters were modeled using the PEP-FOLD3 server [41]. The 3D structures of corresponding HLAs were retrieved from the Protein Databank (PDB) for in silico molecular docking. HAWKDOCK server was used for epitope-HLA docking followed by an MM/GBSA analysis [42]. This is a multi-functional program that has integrated the ATTRACT algorithm for docking, a HawkRank scoring function, and an MM/GBSA free energy decomposition analysis. The scale solvent methods and molecular mechanical energies are combined in the MM/GBSA approach [43]. Because of the convergence issues in some cases, the entropy term was excluded and could not be calculated. The best docking configurations were chosen using the HAWKDOCK’s MM/GBSA scoring function.

### 2.6. mRNA Vaccine Sequence Construction

The presence of five critical components in the Open Reading Frame is required for a highly immunogenic mRNA vaccine formulation: 1. Kozak sequence; 2. Linkers; 3. B and T cell Epitopes; and 4. Stop codon. The AUG codon (the start codon) has to be part of the Kozak sequence [44], whereas the stop codon can be optimized [45]. The selection of linkers that should be flexible, rigid, and cleavable is critical in the development of a multi-epitope vaccine because it allows the domains to operate independently while avoiding contact and interference [46]. Based on previous studies [47,48], we chose the linkers by considering their length and rigidity–flexibility properties. The vaccine sequence was constructed by placing AAY, GPGPG, and KK linkers to connect the MHC I, MHC II, and linear B cell epitopes, respectively [49].

Another sequence that has been previously reported is a signal peptide which helps to increase antigen presentation (to the discharge of translated epitopes that need to leave the cell). As a result, the tissue Plasminogen Activator (tPA) secretory signal sequence was included in the ORF’s 5′ regions [50]. The protein sequence of tPA (UniProt ID: P00750) was obtained from UniprotKB [50]. Since mRNA-based therapeutics are prone to instability, finding the elements in eukaryotic mRNAs was essential [51,52]. As a result, the sequences for the 5′ cap, poly(A) tail, and 5′ and 3′ untranslated regions were required. The length of the poly (A) tail is important because tails that are too long or too short are linked to inefficient translation [53]. We proposed that the poly (A) tail be 115–150 nucleotides long, as this has been suggested as the optimal length by several previous studies [54]. Poly (A) tails have been discovered to work in tandem with 5′ m7G cap sequences [55]. We included the NCA-7d at the 5′ UTR and S27a+R3U as the 3′ UTR regions in our vaccine sequence because they are known to stabilize the mRNAs [56,57].

### 2.7. Immune Simulation

In silico simulation of the vaccine induced immune system response was carried out using the C-ImmSim server [58]. The C-immsim server uses a Position-specific Scoring Matrix (PSSM) and ML to predict the immune system response. Here we set an interval of four weeks of vaccine dose administration as suggested [59]. The simulation was set for three injections, with an interval of four weeks and each dose contained 1000 units of vaccine. The C-ImmSim server sets a time-step scale for simulating an immune response to the vaccine where each time step corresponds to eight hours in real life. We set the total number of steps to 1050 with three injection points at time step 1, 84, and 168. The rest of the parameters were unchanged.

### 2.8. Molecular Simulation

We assessed the stability of the HLA-epitopes by performing an all-atoms simulation using a molecular simulation tool known as AMBER 20. In this regard atom parameterization was achieved through the recruitment of the FF19SB force field. The systems were prepared by adding water molecules and no effect of charge was left. The next phase was to relax each system through energy minimization. In multiple steps the minimization was completed and followed by the heating of each system. The equilibration and production were carried out for each system and a cumulative 100 ns simulation was performed. A post-dynamics assessment of the complex was performed to compute the stability and flexibility using PTRAJ and CPPTRAJ [60,61,62]. 

## 3. Results

### 3.1. Proteins Retrieval for Vaccine Design

Amino acid sequences of the selected proteins (Major caps protein, Envelope glycoprotein M, Envelope glycoprotein H, DNA polymerase processivity factor BMRF1, Protein BDLF2, and Thymine kinase) were retrieved from the Universal proteins knowledgebase (UniprotKB). The Vaxijen server predictions at 0.4 thresholds affirmed that the selected proteins were able to induce a host immune response. The complete information of the selected proteins and corresponding scores are in Table 1.

### 3.2. Prediction and Evaluation of MHC I Epitopes

NetCTL1.2 was utilized for the prediction of MHC I epitopes for chosen EBV proteins. The high combined score of an epitope corresponds to a high binding affinity for MHC-I receptors in terms of vaccine development. The epitopes predicted previously were filtered as per the combined score, immunogenicity > 0, antigenicity ≥ 0.4, solubility > 1, and toxicity < 0. Overall, six MHC I epitopes that were antigenic, immunogenic, soluble in water, non-toxic, and conserved were selected to construct the vaccine sequence. The selected epitopes with corresponding scores are provided in Table 2.

### 3.3. Prediction and Evaluation of MHC II Epitopes

MHC-II binding epitopes activate the helper T cells of the immune system. The IEDB MHC-II module predicted binding of the HTL epitopes corresponding to the reference set of seven human leukocyte antigens: HLA-DRB1*03:01, HLA-DRB1*07:01, HLA-DRB1*15:01, HLA-DRB3*01:01, HLA-DRB5*01:01, HLA-DRB3*02:02, and HLA-DRB4*01:01. MHC II epitopes that have the lowest adjusted percentile rank correspond to a high binding affinity for MHC II molecules. The predicted HTL epitopes were filtered according to the lowest percentile score, antigenicity ≥ 0.4, immunogenicity > 0, solubility > −1, allergenicity < 0.3, interferon-gamma inducing ability, and toxicity < 0. Overall, four HTL epitopes that were antigenic, immunogenic, soluble in water, non-toxic, conserved, non-allergenic, and interferon-inducing were selected for vaccine construction: PTVGMIRDLSASFMT_876–890_ and LERGLINTVLSVKLR_172–186_ (Major Caps Protein), GEVALIKARKKVSGL_98–112_ (Envelope Glycoprotein M), and LMLLRSQLLSYSDFI_404–418_ (Thymine Kinase). The selected HTL epitopes with corresponding scores are provided in Table 3.

### 3.4. Epitopes’ Population Coverage

**Figure 2:**
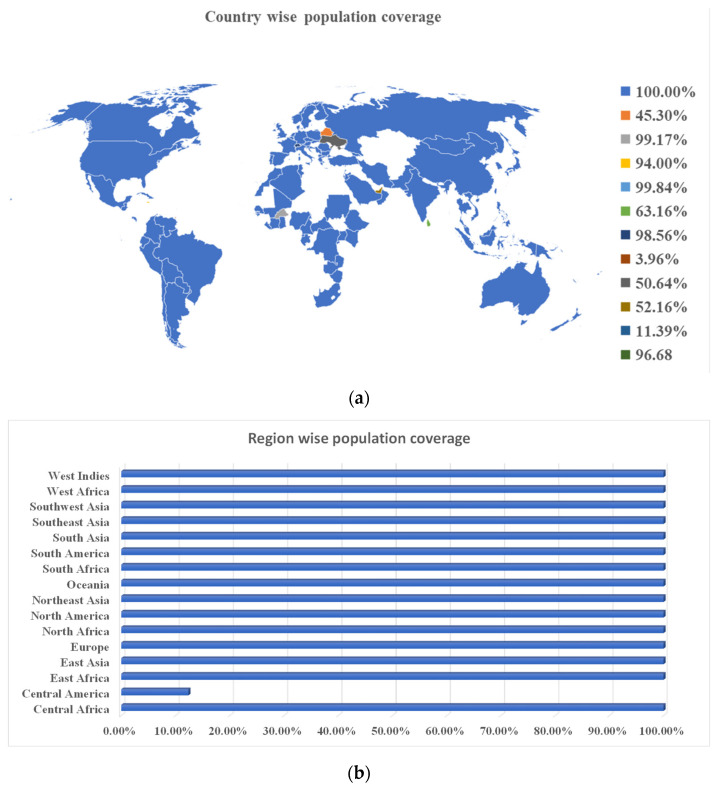
(**a**). Country-wise population coverage for selected epitopes ranges from the lowest 11.39% (Wales) to 100% (Pakistan, Indonesia, China, India, United States of America, and most of the populous countries). (**b**). Region-wise population coverage for the selected T cell epitopes ranging from the lowest 12.38% (Central America) to 100% in all other regions across the globe.

HLA allele diversity and expression differs between ethnicities and geographical regions. As a result, it impacts on the development of an MEV. The IEDB population coverage tool was used to assess the population coverage and suitability of the selected T cell epitopes. The selected epitopes revealed a world population coverage of 99%. The majority of the countries for which data is available showed 100% population coverage. Region-wise, the population coverage analysis revealed 100% population coverage except for Central America, which has the lowest (12.38%) population coverage. The overall population coverage is further depicted in Figure 2.

### 3.5. Prediction and Evaluation of Linear B Cell Epitopes

The BCPreds server predicted linear B cell epitopes for selected EBV proteins. Linear B cell epitopes with higher scores corresponded to the strong binding potential for B cell receptors. The predicted linear B cell epitopes were prioritized according to the high binding score, antigenicity ≥ 0.4, and allergenicity (<0.3). Overall, 10 linear B cell epitopes that qualify for the filtering parameters were selected for constructing the final vaccine. The selected linear B cell epitopes with corresponding scores are provided in Table 4.

### 3.6. Peptide-HLAs Molecular Docking

Six CTL and four HTL epitopes were modeled for in silico molecular docking using PEP-FOLD3. T cell epitopes along with corresponding HLAs are listed in Table 5. Three-D models of eight HLAs were downloaded from PDB and prepared for docking: HLA-B*15:01 (5V4M), HLA-A*02:06 (3OXR), HLA-A*01:01 (4NQX), HLA-A*02:01 (4L29), HLA-DRB3*01:01 (2Q6W), HLA-DRB1*07:01 (6BIJ), HLA-DRB5*01:01 (1H15), HLA-DRB1*15:01 (1BX2). The modeled 3D structures of respective HLAs, the validation reports, and interactions are shown in Supplementary Figures S1–S3.

**Figure 3:**
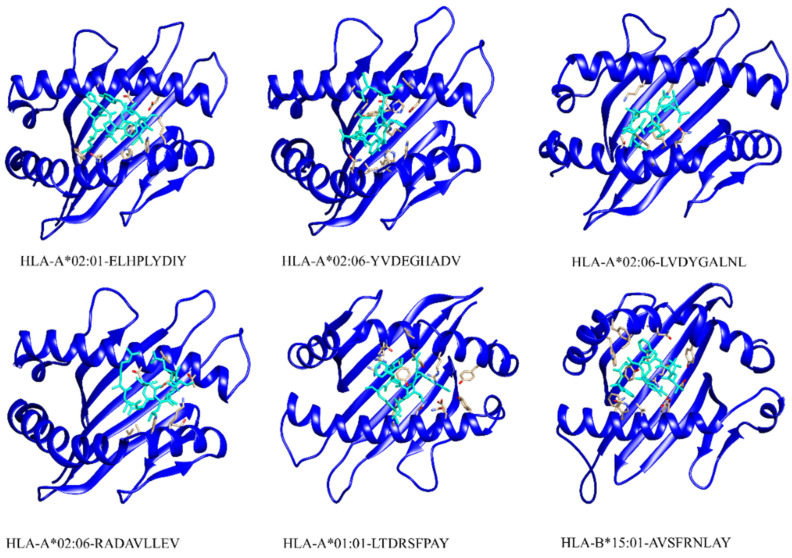
Selected CTL epitopes’ docking complexes with respective HLAs. The epitope is shown in a cyan color whereas the human leukocyte antigens are presented in a dark blue color.

**Figure 4:**
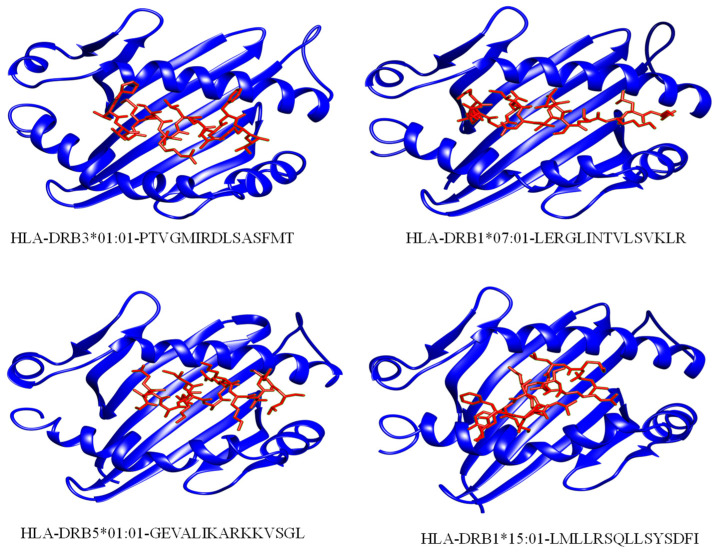
Selected HTL epitopes’ docking complexes with respective HLAs. The epitope is shown in a red color whereas the human leukocyte antigens are presented in a dark blue color.

**Figure 5:**
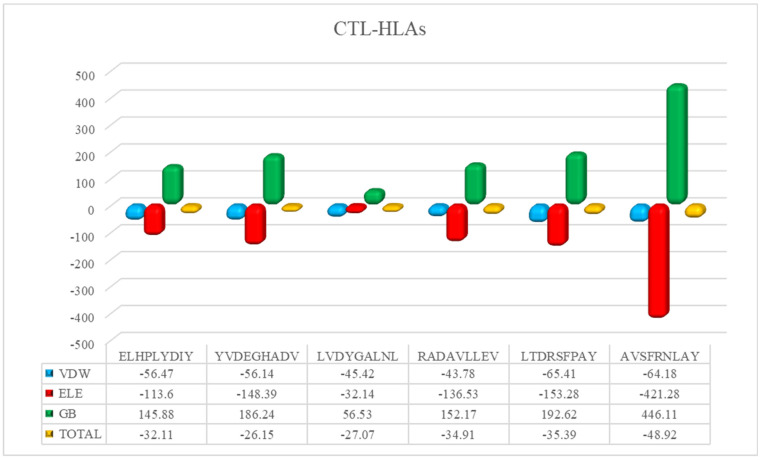
The vDW energy, Electrostatic, and Binding free energy for selected CTL epitopes in complex with respective HLA molecules.

**Figure 6:**
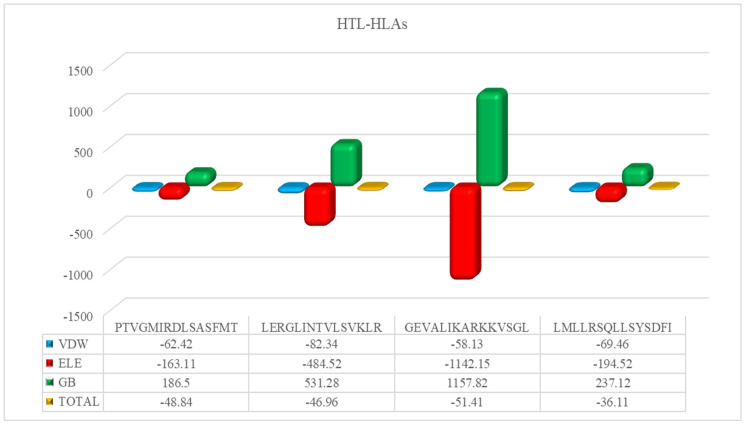
The vDW energy, Electrostatic, and Binding free energy for selected HTL epitopes in complex with respective HLA molecules.

The HAWKDOCK server predicted the stable conformation of the modeled peptide into the binding groove of the respective HLA receptor [42]. The CTL epitopes’ docking scores with corresponding HLAs are as follows: total binding free energy for HLA-A*02:01-ELHPLYDIY was reported to be −32.11 kcal/mol, −26.15 kcal/mol for HLA-A*02:06-YVDEGHADV, −27.07 kcal/mol for HLA-A*02:06-LVDYGALNL, −34.91 kcal/mol for HLA-A*02:06-RADAVLLEV, −34.91 kcal/mol for HLA-A*01:01-LTDRSFPAY, and −48.88 kcal/mol for HLA-B*15:01-AVSFRNLAY. Whereas, for the docking complexes of HTL-HLAs, the total binding free energies were: −46.96 kcal/mol for HLA-DRB1*07:01-LERGLINTVLSVKLR, −51.41 kcal/mol for HLA-DRB5*01:01-GEVALIKARKKVSGL, −36.11 kcal/mol for HLA-DRB1*15:01-LMLLRSQLLSYSDFI, and −48.4 kcal/mol for HLA-DRB3*01:01-PTVGMIRDLSASFMT. The CTL–HLA and HTL–HLA docking complexes are presented in Figure 3 and Figure 4, respectively. However, Figure 5 and Figure 6 have the graphical description of the docking results.

### 3.7. mRNA Vaccine Construction

**Figure 7:**
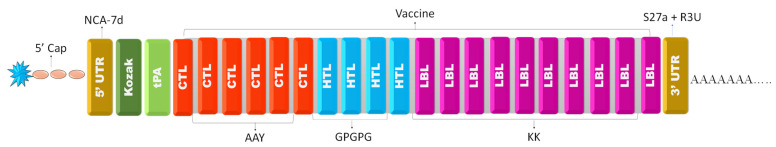
The final vaccine construct, blue with light Pink (5′ cap), brown (5′ UTR and 3′ UTR), light-dark green (Kozak Sequence), light green (Signal proteins), red (CTL epitopes), blue (HTL epitopes), purple (LBL epitopes), light gray (EAAAK, AAY, GPGPG, and KK linkers), and gray (Poly A tail).

The final vaccine construct comprised six CTL epitopes (two epitopes from the gpM and major caps protein, each, and one epitope from the gpH, BDLF2. BMRF1, and thymidine kinase, each), three HTL epitopes (two epitopes from the major caps protein and one epitope from the gpM, gpH and thymidine kinase, each) and 10 linear B cell epitopes (two epitopes from the major caps protein, gpH, thymidine kinase and BMRF1, each and one epitope from the BDLF2 and gpH, each). The final CTL, HTL, and LBL epitopes were joined by AAY, GPGPG, and KK linkers, respectively. The final mRNA vaccine started from tPA and ended at the stop codon added right before a 3′ UTR region, namely S27a+R3U and a Poly (A) tail of 120 nucleotides at the end. The CTL, HTL, and LBL epitopes included in the final construct are listed in Table 2, Table 3 and Table 4, respectively. The mRNA vaccine is graphically presented in Figure 7 with the components in the same order as placed in the actual vaccine construct.

### 3.8. Immune Simulations

**Figure 8:**
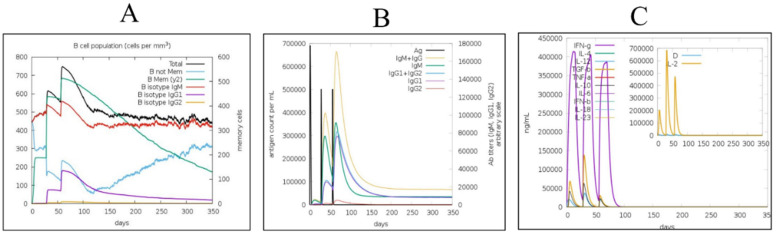

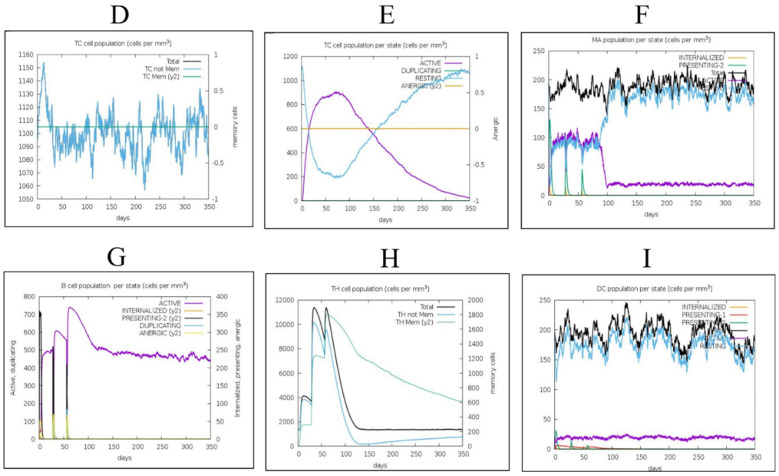
Simulations of the host immune response upon introduction of the vaccine designed. (**A**) B cell population after three injections, (**B**) immunoglobulin production in response to the injection of antigens to the host body, (**C**) Interleukin and cytokine levels, (**D**) Cytotoxic T cell population, (**E**) Cytotoxic T cell population per state, (**F**) Macrophage population per state, (**G**) B cell population per state, (**H**) Helper T cell population, and (**I**) Dendritic cell population per state.

The secondary response reported in the simulations was noted to be higher than the primary, and similarly for the tertiary immune response. The immune simulations results were as expected and are presented in Figure 8. The immunoglobulin (Ig) M concentration was higher than that of IgG. Both the secondary and tertiary immune system responses showed high levels of immunoglobulin response, as expected, with a simultaneous reduction in antigen level. This specifies the formation of an immune system memory induced by the vaccine, and as a result, an effective antigen immunity in future antigen exposures. Furthermore, it was shown that some B-cell isotypes may persist for a long period, suggesting that isotype flipping and memory formation are possible. The CTL and HTL populations exhibited a similar rise in response to their respective memory development. Macrophage activity increased while the activity of dendritic cells remained very stable during the simulation time. The IFN- and IL-2 levels were increased as well. In addition, mechanisms of the innate part of the immune system (for example the epithelial cells) were activated. A lower Simpson index (D) showed the likelihood of a wide diversity of the immune responses.

### 3.9. Molecular Simulation Analysis

**Figure 9:**
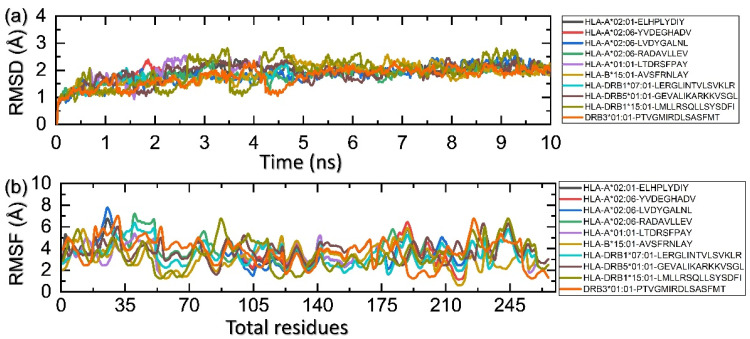
Calculation of RMSD (**a**) and RMSF (**b**) for each HLA and epitope.

The assessment of molecular stability of each complex during the simulation demonstrates essential knowledge regarding the stable interaction of biological molecules. It yields significant information about the inhibition and activation pathways. Hence, the significance of this approach was considered, and the stability was calculated as root mean square deviation (RMSD). Figure 9a shows that each complex achieved the stability at 2 Å and the average RMSD was reported to be 1.80 Å. This shows that all the epitopes bind to their respective stability to induce an immune response. On the other hand, residual fluctuation, which is considered essential for deciphering knowledge about the functionality of protein, was also calculated as root mean square fluctuation (RMSF). The RMSF pattern was observed to be alike in minimal regions while different fluctuations were maximum. This demonstrates that the interaction of each epitope produces a differential fluctuation effect, which consequently alters the dynamic properties of each allele. The RMSF of each complex is shown in Figure 9b.

## 4. Discussion

Efforts to reduce EBV’s pathogenic activity have been persistent, necessitating a variety of therapeutic strategies ranging from small molecule inhibitor development to immunization. Vaccines containing lytic and latent EBV proteins are being developed to evoke an immune response [71]. Many attempts to develop an EBV vaccine have failed; in clinical trials, a gp-350-based vaccine made substantial progress, although it was unable to prevent EBV infections [14,71]. Of all the different types of vaccines, mRNA vaccines have risen as a promising preventive option because they may be designed using critical information from viral pathogenic components. mRNA vaccines, like those developed against the Zika virus, HIV-1, influenza virus, rabies, and other viruses, have represented a diverse and highly efficient subgroup of vaccine candidates from the first successful example of mRNA treatments in 1990. Despite the obstacles such as mRNA instability due to widespread RNase degradation and inherent immunogenic ability due to detection by the innate immune system [13,15,20,72], significant progress in mRNA vaccine development has been made. Such vaccines can provide protection against pathogens that lasts for many years. For any vaccination strategy, it is critical to induce both B and T cells to facilitate the immune response because it results in a more efficient and robust immune response when the target pathogen is encountered by the host body [20]. A vaccine for EBV disease must also meet the following criteria, which are similar to what is being proposed for the ideal vaccine: (i) It must be reasonably safe; (ii) It must induce fairly long protection against the large diversity of EBVs; (iii) Protection must be population-wide and should be actually accomplished with the least number of vaccinations; (iv) It must be formulated, developed and produced very cost-effectively; (v) It ought to be efficacious prophylactically and preventatively. Reverse vaccinology has been established as a solution for the shortcomings of traditional vaccinations. As we learn more about the human immune system, vaccine development has accelerated. The development of immunizations has dramatically improved human health and life expectancy around the globe. Through immunization, many lives have been saved, lowering medical expenses and raising living standards. However, the effects of conventional vaccination designs on human immune systems still need to be better understood. Regarding newly emerging human viral infections, several immune-related aspects need to be considered. Advanced reverse vaccinology techniques enable targeted adaptive immune response activation. Using the epitope prediction method, several vaccines have been developed to protect against human illnesses. This has entailed developing a potential vaccine candidate based on the epitope (AMA-1) against “Plasmodium vivax”. A recently developed multi-epitope vaccine with a strong IgG antibody-specific immunological response against “Acinetobacter baumannii” was evaluated in mice in 2019. Additionally, it was confirmed that theoretically anticipated B cell epitopes are efficient at detecting “Trypsonoma vivax”. Examples of deadly human viruses that have been combated with comparable tactics, include the Mokola Rabies virus, the Crimean-Congo hemorrhagic fever virus, the Marburg virus, and the Ebola virus [73,74,75].

TCRs identify antigens displayed on the surface of APCs that have MHC receptors (classified as class I and II) on their surface. These antigens are recognized by the cytotoxic and helper T-cells, respectively. BCRs bind antigens from foreign particles and induce B cell maturity and differentiation that leads to the release of antibodies [50].

For instance, computational biology helps to unveil the molecular mechanism and discovery of new drugs, vaccines, or peptides for different pathogens [76,77,78,79,80]. Epitope-based mRNA vaccines developed using computational biology approaches have addressed some of the aforementioned characteristics since the very beginning of the development process. The computer-guided design of vaccines has a number of benefits, but one drawback of our method was that it could only predict linear epitopes, which leaves out the conformational ones from the assembly. In this scientific study, immunoinformatics tools were utilized to predict B and T cell epitopes that are specifically presented to CTL, HTL, and B cells, and these epitopes were derived from six antigenic proteins of EBV that play a key role in the attachment and infection of the viral particles. The inclusion of residues with large, hydrophilic, and aromatic side chains can reveal information about the predicted epitope’s immunogenicity and surface exposure [81]. The immune response to viral infections depends highly on CTL-mediated cytotoxicity. Some viral proteins are degraded by virus-infected cells and presented to CTLs with MHC class I molecules. The CTLs kill the infected cells by releasing cytotoxic granules after recognizing degraded regions of viral proteins termed epitopes [82]. Eight CTL epitopes as mentioned in the result section, were used for vaccine construction in this study. The HTLs were activated when the APCs present viral particles to the HTLs along with the MHC class II molecules. The HTLs release a variety of cytokines and chemokines in response to epitope identification, including IFN-, IL-4, and IL-10, which play different roles in the immune response against invaders [83,84,85]. Five HTL epitopes fulfilling the set parameters were selected for vaccine construction in this study. Most effector T lymphocytes (CTLs and HTLs) perish, once antigens are cleared, but a small percentage survive to form the memory T cell reservoir [86,87]. B lymphocytes have membrane-bound immunoglobulins that bind to epitopes on the surface of target cells, internalize them, process them, and deliver them to the T cells via B cell receptors [88]. These processed epitopes are presented on the surface of B cells with MHC class II and are recognized by the HTLs with a corresponding T cell receptor. This causes B lymphocytes to differentiate into antibody-producing plasma cells [89,90]. These antibodies play an important role in pathogen neutralization [91]. On the other hand, activated B lymphocytes trigger the formation of long-lived plasma cells and memory B cells [92]. Ten linear B cell epitopes fulfilling the set parameters were selected in this study. Molecular docking is a popular bioinformatics approach for predicting the binding affinity and position of a ligand and corresponding receptor. It was used to simulate the binding affinity of T cell epitopes for their corresponding MHC molecules. The energy generated during spontaneous bond formation between a receptor and its ligand can be used to determine binding affinity. The lower the energy, the more strongly the receptor is bound to its ligand [93]. The selected T cell epitopes fulfilling all the parameters were modeled for molecular docking using the pepfold3 server, whereas the PDB files of corresponding HLAs were retrieved from the protein data bank. In silico molecular docking of ten T lymphocyte epitopes with corresponding HLAs were carried out using Hawkdock server. Furthermore, the docking poses for each were chosen based on the least MM/GBSA scores.

Each of the epitopes in our devised method, derived from various antigens, represents a distinct immunological component. The position and order of the T and B cell epitopes within the construct will need to be carefully considered because the final vaccine must incorporate both. The epitopes were arranged in “string-of-beads” design as presented in Figure 7. The importance of carefully selecting the spacers lies in their potential to be immunogenic or efficiently separate novel epitopes that would induce the desired immune response. We have incorporated AAY, GPGPG, and KK linkers to link CTL, HTL, and LBL epitopes, respectively, the arrangement of epitopes in the vaccine constructed in this study is presented in Figure 7. However, the final localization and ordering of the epitopes and any interstitial spacers will require empirical determination. 

Likewise, even though the final construct’s VaxiJen v2 score was over the cut-off, their overall immunogenicity will need to be carefully investigated experimentally since the immunogenicity of isolated epitopes may vary when they have been joined in a string-of-beads array. Optimal spacers with a balance of flexibility and rigidity prevent inter-domain interactions and promote the mRNA vaccine’s proper functioning. Several studies have reported that poly(A) tails function synergistically with 5’ m7G cap sequences, with the optimal length of 120–150 base pairs. The mRNA ORF must be flanked by the 5’ and 3’ UTRs to enhance translation and stability [94]. NCA-7d was added as the 5’ untranslated region (UTR), and S27a+R3U as the 3’ UTR region to the mRNA vaccine sequence since they are known to stabilize mRNAs. The sequences around the stop codon could be improved and attention should be given to the Kozak sequence [94]. The inclusion of secretory signal sequences, and those that contain directed information about specific endoplasmic reticulum compartments (e.g., MHC I), has been linked to increased vaccine efficacy in mRNA and DNA-based vaccines [95,96].

In silico immune responses, following repeated antigen exposure showed an overall rise in immunogenicity. The presence of humoral immunity, which is essential for a full immune response, was shown by increased B and T cell activity and also B cell memory that persisted for several months. High levels of IFN- and IL-2 release upon constant exposure suggested a cell-mediated immune response. An immune response can also be boosted by cytokine IFN-, which promotes B cell proliferation and isotype switching [97,98]. The activities of dendritic cells and macrophages, and the Simson index, were found to be satisfactory. This vaccine profile suggests immune memory formation and natural immune protection against EBV.

## 5. Conclusions

The recent shift of interest from conventional to mRNA vaccines has proven its usefulness. The mRNA vaccine is able to induce a proper immune response towards the subject pathogen and provide long-lasting immunity. Despite no major advances to date, vaccinations remain a potential therapeutic strategy for suppressing the virulence of the Epstein-Barr virus, which is linked to infectious mononucleosis and various cancers in humans. In our study we have used an immunoinformatic and structural bioinformatics approach to rationally develop an mRNA vaccine for immune protection against EBV. The selection of candidate proteins and prioritization of epitopes were based on tested protocols. Moreover, T cell epitopes, both CTL and HTL, were predicted using the NetCTL1.2 and IEDB MHC-II webservers, respectively. Peptide modeling and in silico molecular docking were utilized to analyze the binding potential of selected T cell epitopes for corresponding HLA molecules. An advantage of the immunoinformatic over the conventional laboratory approaches is the low cost, quick identification, and facility for screening epitopes within the vaccine design process. Furthermore, to validate the outcomes of this in silico study, experimental studies are required to validate the effectiveness of vaccines constructed against EBV.

## Figures and Tables

**Table 1:** Selected proteins with respective antigenic scores predicted by the Vaxijen server.

S.NO	Protein	UniProt ID	Antigenic Score	Amino Acid Count	Function	Reference
1	Major capsid protein	P03226	0.42	1381	Major Capsid Protein plays a key role in capsid self-assembly of EBV.	[63,64]
2	Envelope glycoprotein M	P03215	0.48	405	Viral self-assembly. It also aids gpL and gpH in correctly incorporating into the virus membrane.	[65]
3	Envelope glycoprotein H	P03231	0.53	706	It aids viral entry into host cells.	[66]
4	DNA polymerase processivity factor BMRF1	P03191	0.6	404	It has a critical role in lytic DNA replication. It also Interacts with DNA binding proteins of EBV.	[67,68]
5	Protein BDLF2	P03225	0.52	420	Interacts with protein BMRF2, a key role in the rearrangement of cellular actin to promote cell-to-cell of EBV in the epithelial cells.	[64]
6	Thymidine kinase	P03177	0.4	607	Activates and re-activates the replication of viral DNA.	[69,70]

**Table 2:** Selected CTL epitope with respective binding affinity and physiochemical characteristics. Antigenic (>0.4), Immunogenic (<1), Soluble (>1), and Non-Toxic (<0).

Protein	ID	Peptide	Comb	Antigenicity	Immunogenicity	Solubility	Toxicity
Major caps protein	541	ELHPLYDIY	0.95	1.05	0.08	1.24	−0.88
	799	YVDEGHADV	0.95	0.43	0.21	1.91	−0.69
Envelope glycoprotein m	49	LVDYGALNL	0.8	1.24	0.15	1.32	−0.68
Thymine kinase	534	RADAVLLEV	0.94	0.6	0.12	1.39	−0.94
Protein bdlf2	402	LTDRSFPAY	3.36	0.97	0.02	1.53	−1.1
DNA polymerase processivity factor BMRF1	83	AVSFRNLAY	2.25	1.71	0.12	1.36	−1.33

**Table 3:** Selected HTL epitopes with respective binding affinity and physiochemical properties. Antigenic (>0.4), IFN gamma inducing ability, non-Allergenic (<0.3) Soluble (>1), and Non-Toxic (<0).

Proteins	Start–End	Peptide	Percentile Rank	Antigenicity	IFN Epitope	Allergenicity	Solubility	Toxicity
Major Caps Protein	876–890	PTVGMIRDLSASFMT	0.51	0.43	Positive	0.27	1.22	−0.95
	172–186	LERGLINTVLSVKLR	0.78	0.73	Positive	0.3	1.07	−1.42
Envelope Glycoprotein M	98–112	GEVALIKARKKVSGL	0.04	0.93	Positive	0.3	1.93	−1.35
Thymine Kinase	404–418	LMLLRSQLLSYSDFI	0.77	0.63	Positive	0.23	1.16	−1.81

**Table 4:** Selected LBL epitopes with corresponding scores. Antigenic (>0.4) and non-Allergenic (<0.3).

Protein	ID	Peptide	Score	Antigenicity	Allergenicity
Major caps protein	475	NAAPAPRDRRETYSLQHRRP	0.99	1.35	0.28
	638	PLVSLCINTYWERSGRLAFV	0.96	0.7	0.26
Envelope glycoprotein M	193	FLWWVVFYLKPVVTNLYLGC	0.82	0.92	0.3
Envelope glycoprotein H	131	FYYIGTMLPNTRPHSYVFYQ	0.94	0.5	0.28
	272	LEMKGGCREPELDTETLTTM	0.91	0.66	0.29
Thymine kinase	89	AVTSNTGNSPGSRHTSCPFT	1	0.5	−0.2
	199	HSALKQKNGGKGKPSGLFEH	0.99	0.64	−0.1
Protein BDLF2	117	EMDDTMASSGGQRGAPISAD	0.95	0.72	0.29
DNA polymerase processivity factor BMRF1	380	KRTSSEARQKQKHPKKVKQA	0.96	0.72	0.25
	146	MPYMPPASDRLRNEQMIGQV	0.77	0.48	0.3

**Table 5:** Selected T cell epitopes, HLA molecules, and binding affinity towards the respective receptor.

Protein	HLA	PDB ID	Peptide	Affinity	MHC
Major Caps Protein	HLA-A*02:01	4L29	ELHPLYDIY	0.57	MHC-I
	HLA-A*02:06	3OXR	YVDEGHADV	0.75	MHC-I
Envelope Glycoprotein M	HLA-A*02:06	3OXR	LVDYGALNL	0.22	MHC-I
Thymine Kinase	HLA-A*02:06	3OXR	RADAVLLEV	0.55	MHC-I
Protein BDLF2	HLA-A*01:01	4NQX	LTDRSFPAY	0.99	MHC-I
DNA Polymerase Processivity Factor BMRF1	HLA-B*15:01	5V4M	AVSFRNLAY	0.7	MHC-I
Major Caps Protein	HLA-DRB3*01:01	2Q6W	PTVGMIRDLSASFMT	0.51	MHC II
	HLA-DRB1*07:01	6BIJ	LERGLINTVLSVKLR	0.78	MHC II
Envelope Glycoprotein M	HLA-DRB5*01:01	1H15	GEVALIKARKKVSGL	0.04	MHC II
Thymine Kinase	HLA-DRB1*15:01	1BX2	LMLLRSQLLSYSDFI	0.77	MHC II

## Data Availability

The data presented in this study are available within the article.

## Supplementary Materials

The following supporting information can be downloaded at: https://www.mdpi.com/article/10.3390/ijerph192013054/s1, Figure S1. The 3D model of HLA-A*30:02 generated by Swiss model server and its validation by ERRAT, Prosa-Web and Procheck. (A) the 3D model of HLA molecule (B) The ERRAT score of 99.41 (C) Prosa-Web quality score −7.28 and (D) Ramachandran plot by Procheck shows 91.2% residues in most favored region, 8.1% in additional allowed region, 0.6% in generously allowed region and 0.6% in disallowed region. Figure S2. The 3D model of HLA-DRB1*03:01 generated by Swiss model server and its validation by ERRAT, Prosa-Web and Procheck. (A) the 3D model of HLA molecule (B) The ERRAT score of 97.2 (C) Prosa-Web quality score −5.52 and (D) Ramachandran plot by Procheck shows 90.4% residues in most favored region, 9% in additional allowed region, none of the residues in generously allowed region and 0.6% in disallowed region. Figure S3. Selected HTL epitopes docking complex with respective HLAs. The epitope is shown in red color whereas the human leukocytes antigens are presented in dark blue color.
