# Supplementary material for: mRNA-Based Vaccine Designing against Epstein-Barr Virus to Induce an Immune Response Using Immunoinformatic and Molecular Modelling Approaches
Source: Int J Environ Res Public Health. 2022 Oct 11;19(20):13054. doi: 10.3390/ijerph192013054 (PMC9602923; doi:10.3390/ijerph192013054)
Supplement: Supplementary file 1 [file ijerph-19-13054-s001.zip › ijerph-1881217-supplementary.pdf]

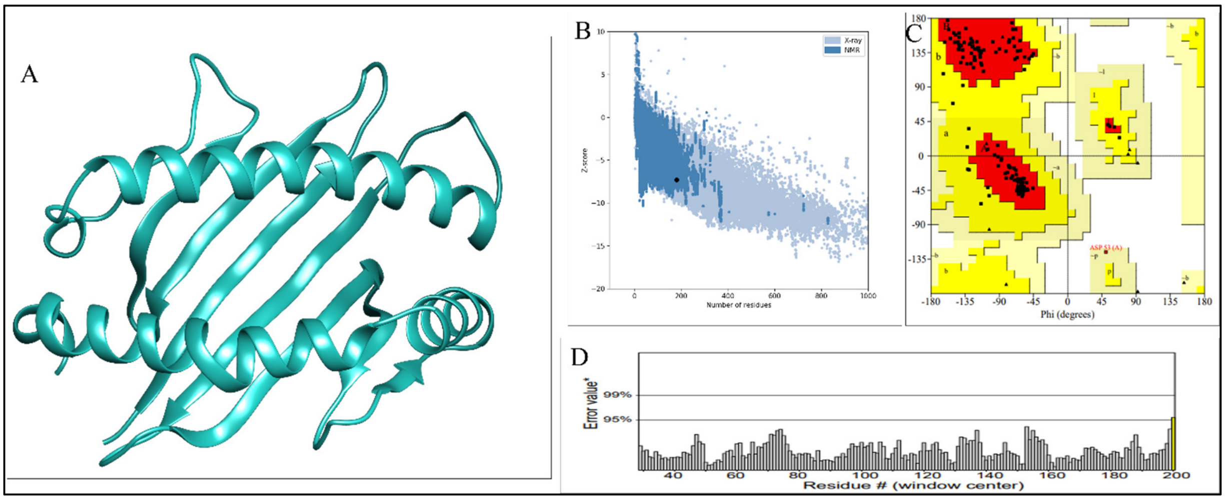


**Figure S1**. The 3D model of HLA-A*30:02 generated by Swiss model server and its validation by ERRAT, Prosa-Web and Procheck. (A) the 3D model of HLA molecule (B) The ERRAT score of 99.41 (C) Prosa-Web quality score -7.28 and (D) Ramachandran plot by Procheck shows 91.2% residues in most favored region, 8.1% in additional allowed region, 0.6% in generously allowed region and 0.6% in disallowed region

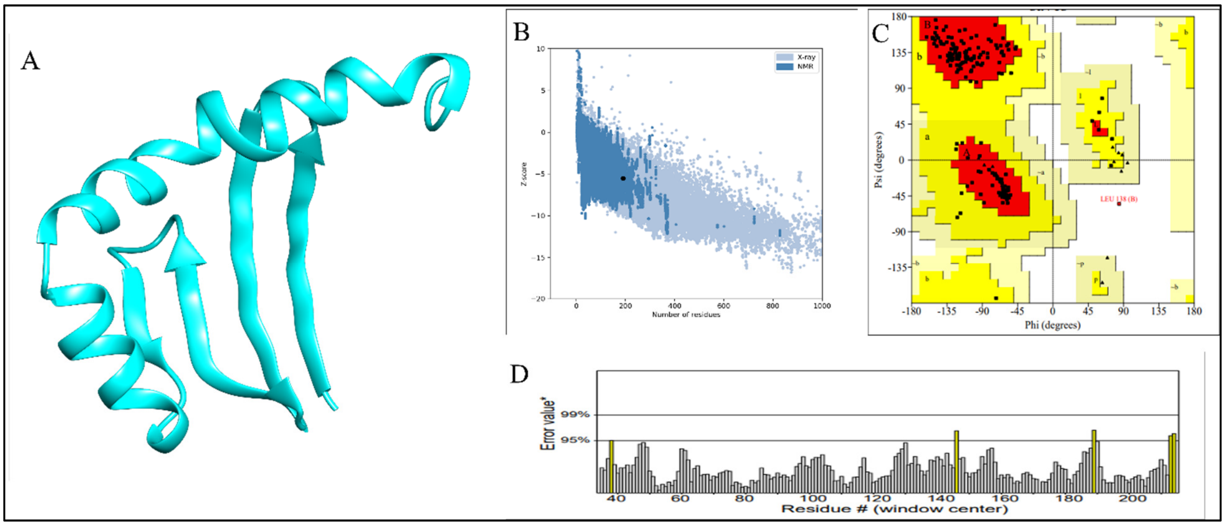


**Figure S2**. The 3D model of HLA-DRB1*03:01 generated by Swiss model server and its validation by ERRAT, Prosa-Web and Procheck. (A) the 3D model of HLA molecule (B) The ERRAT score of 97.2 (C) Prosa-Web quality score -5.52 and (D) Ramachandran plot by Procheck shows 90.4% residues in most favored region, 9% in additional allowed region, none of the residues in generously allowed region and 0.6% in disallowed region.

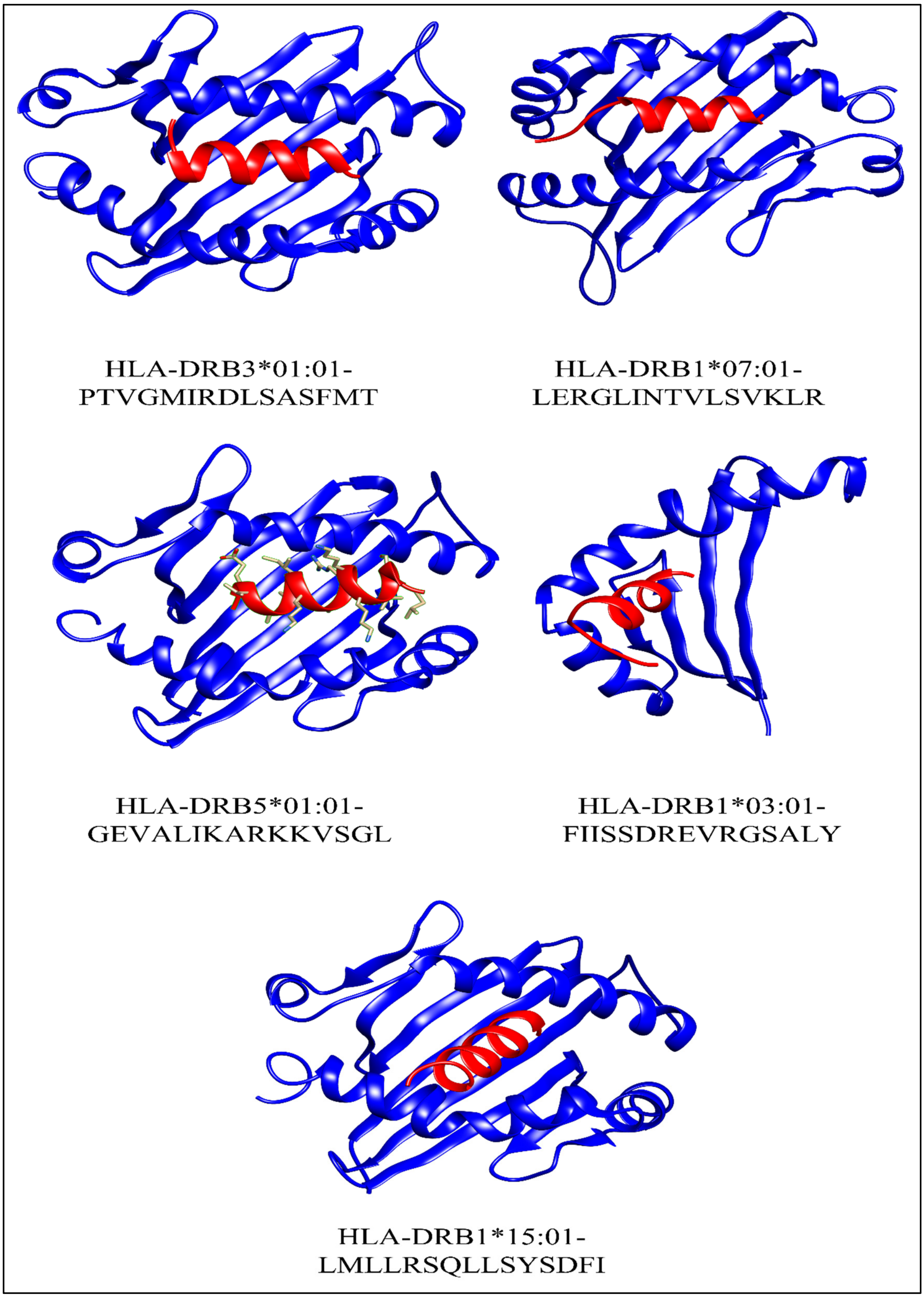


**Figure S3.** Selected HTL epitopes docking complex with respective HLAs. The epitope is shown in red color whereas the human leukocytes antigens are presented in dark blue color.
